# Prospective Assessment of Gonadotropin Therapy and Its Outcome in Nonobstructive Azoospermic Patients

**DOI:** 10.7759/cureus.84071

**Published:** 2025-05-14

**Authors:** Ahsan Ahmad, Bhramita Roy, Gaurav Babelay, Kumar Dheeraj

**Affiliations:** 1 Department of Urology, Indira Gandhi Institute of Medical Sciences, Patna, IND; 2 Department of Reproductive Medicine, Indira Gandhi Institute of Medical Sciences, Patna, IND

**Keywords:** azoospermia, infertility, male, nonobstructive azoospermia, spermatozoa

## Abstract

Background

The absence of spermatozoa in the ejaculate after centrifugation is known as azoospermia. The primary purpose of the study was to determine the recombinant gonadotropin therapy on stimulating spermatogenesis in nonobstructive azoospermia (NOA) patients having spermatogenic failure. Additionally, the treatment's clinical outcome and potential adverse effects were evaluated.

Materials and methods

It was a prospective single-arm observational study and included 20 patients. The study is conducted at the Department of Urology and Infertility Clinics, Indira Gandhi Institute of Medical Sciences (IGIMS), Patna, Bihar, India. It lasted over the period from November 2023 to December 2024. The institutional ethics committee (IEC) of IGIMS granted ethical approval under letter number 1271/IEC/IGIMS/2023, dated 05 October 2023.

Results

The mean age of the patients in our study was 31.45 ± 4.82 years. The mean initial follicle-stimulating hormone (FSH) level was 16.69±6.02 IU/l, the mean initial luteinizing hormone (LH) was 9.85 ± 1.95 IU/l, and the mean testosterone level at baseline was found to be 9.95 ± 2.54 nmol/l. The association of sperm extraction with late maturation arrest compared to other histopathological findings was found to be statistically significant with a p-value of <0.001.

Conclusion

It was concluded in the study that gonadotropin therapy is helpful in very selected cases of nonobstructive azoospermia. For men with NOA and spermatogenic failure, recombinant gonadotropin therapy appears to be a useful method of overcoming infertility.

## Introduction

The absence of spermatozoa in the ejaculate after centrifugation is known as azoospermia [1]. It affects roughly 10-15% of infertile males, and generally, infertility is due to this [1,2]. Nonobstructive azoospermia (NOA), a condition characterized by poor spermatogenesis, accounts for around 60% of all azoospermia cases. On the other hand, obstructive azoospermia occurs in 40% of cases and is caused by ductal system blockage [2,3].

For couples pursuing motherhood, NOA is a difficult and upsetting circumstance. The majority of males, particularly in nations with strong cultural and religious values, choose to use their own gametes and reject the possibility of sperm donation [4].

In certain males, residual production of sperm can be detected in the testicular tissue, so it cannot be determined that there is a lack of spermatogenesis if, in any case, sperm is absent in the ejaculate [2,5]. Usually, nonobstructive azoospermia is thought to be incurable. However, new research suggests that exogenous gonadotropins and selective estrogen receptor modulators may accelerate sperm maturation [5]. Compared to individuals with obstructive azoospermia, NOA patients with hypogonadotropic hypogonadism have more interstitial testicular lesions without Leydig cells and fibrosis. Another study by Oka et al. found a correlation between the usage of human chorionic gonadotropin (HCG), a decrease in interstitial lesion, and hypertrophy of Leydig cells, which may boost the rate of sperm retrieval [6].

The primary purpose of the study was to determine the recombinant gonadotropin therapy for stimulating spermatogenesis in NOA patients having spermatogenic failure. Additionally, the treatment's clinical outcome and potential adverse effects were evaluated.

## Materials and methods

Study design

This prospective, single-arm observational study was carried out at the Indira Gandhi Institute of Medical Sciences (IGIMS) in Patna, Bihar, India, at the Department of Urology and Infertility clinics. The study duration was one year and two months, i.e., from November 2023 to December 2024.

Study population

Overall, 20 patients were part of the study. A convenient sampling technique was used. The inclusion criteria for enrollment of participants were male individuals between the ages of 18 and 45 with NOA diagnosed clinically on the basis of semen analysis. Additionally, patients with genital tract anatomical anomalies, medication allergies, concomitant conditions, and mental illness were excluded from the study.

Study procedure

Treatment was started with an injection of human chorionic gonadotropin (hCG) 1500-2000 IU subcutaneously/intramuscularly twice weekly. The HCG dosage was changed to keep endogenous testosterone levels between 17 and 29 nmol/l (500 and 800 ng/dl) for at least three months and up to nine months. Follicle-stimulating hormone (FSH), luteinizing hormone (LH), testosterone, and estrogen levels were measured every four to six weeks, and recombinant FSH (75-150 IU) twice weekly was added if FSH levels dropped to less than 1.5IU/L. An aromatase inhibitor (anastrozole 1mg/four times a day) was proposed to be added if the testosterone/estradiol (T/E) level was less than 10, but in our study, this ratio was not less than 10, so anastrozole was not added in any cases. Three months following the start of treatment, and thereafter at intervals of four to six months, semen analysis was performed. Thus, after treatment, follow-up was done for six months.

Data collection

Data that were collected during the study were the age of the male partner, genital examination by clinical and ultrasound of the testis, epididymis, vas deferens, seminal vesicle (SV), and varicocele. Baseline semen analysis and an endocrine workup for luteinizing hormone (LH), follicle-stimulating hormone (FSH), total and free testosterone, and estrogen were done. A genetic study for karyotype and chromosome (Chr) Y microdeletion was also done. Testicular fine needle aspiration cytology (FNAC) to look for hypo spermatogenesis, maturation arrest, Sertoli cell only, tubular atrophy, and the mixed pattern was done as part of the work, and then patients were started on hormonal treatment.

Statistical analysis

Data was entered in Excel (Microsoft, Redmond, Washington) and was imported to Statistical Package for the Social Sciences (SPSS) version 26.0 software (IBM Inc., Armonk, New York) for analysis. Whereas continuous variables are described as mean with standard deviation (SD) or median and range, categorical variables are displayed as frequencies and percentages. Results are presented with the help of tables and figures. To compare categorical outcomes, Fisher's exact test was used. An independent t-test was employed for a continuous outcome. The p-value was considered significant at less than 0.05.

Ethical clearance

The Institutional Ethics Committee (IEC) of IGIMS, Patna, Bihar, India, granted ethical approval under letter number 1271/IEC/IGIMS/2023, dated 05 October 2023.

## Results

A total of 20 male patients with NOA were studied. The patients in our study were 31.45 ± 4.82 years old on average. The mean initial FSH level was 16.69±6.02 IU/l, the mean initial LH was 9.85 ± 1.95 IU/l, and the mean Testosterone level at baseline was found to be 9.95 ± 2.54 nmol/l. Table 1 represents a summary of baseline continuous variables.

**Table 1:** Summary of baseline continuous variables Data was presented as mean±SD FSH - follicle-stimulating hormone; LH - luteinizing hormone

Variable	Value
Age (in years)	31.45±4.82
FSH initial (IU/l)	16.69±6.02
LH initial (IU/l)	9.85±1.95
Testosterone (nmol/l)	9.95±2.54

In all patients, NOA was confirmed by preliminary FNAC. Only eight patients (40%) had Sertoli cells, five patients (25%) had late maturation arrest, four patients (20%) had early maturation arrest, and three patients (15%) had hypo-spermatogenesis, according to FNAC. Normal male karyotype (46/XY) was found in 16 patients, while the remaining four (20%) were found to have 46/XXY karyotype. No Y chromosome microdeletion was found in 14 (70%), while six patients had Y chromosome microdeletions, amongst which three (15%) patients had AZFa+ and the remaining three (15%) patients showed AZFc+ microdeletion. Table 2 describes a summary of baseline categorical variables.

**Table 2:** Summary of baseline categorical variables Data was presented as n (%) FNAC - fine needle aspiration cytology; XY - male sex; XXY - Klinefelter syndrome; AZF - azoospermic factors

Variable	Value
Testicular FNAC	
Early maturation arrest	4 (20%)
Late maturation arrest	3 (15%)
Hypospermatogenesis	5 (25%)
Sertoli cell only	8 (40%)
Karyotype	
46/XY	16 (80%)
46/XXY	4 (20%)
Y chromosome microdeletion	
AZFa+	3 (15%)
AZFc+	3 (15%)
None	14 (70%)

The treatment characteristics and outcomes of each patient are presented in Table 3. Hormonal therapy was administered to all patients for a median of nine months. Recombinant HCG was given twice a week. The initial dose of 2000 IU/l was given in all patients, and the final dose reached 5000 IU/l in all but one patient (case no. 1). In case no. 1, a final dose of 4000 IU/l was given. The serum FSH level was supplemented with a recombinant FSH injection when it fell below 1.5 IU/l. This was needed in two patients in our study, i.e., in case 1 and case 9, in whom an initial dose of 75 IU/l was given and a final dose of 150 IU/l was administered. The testosterone to estradiol ratio (T: E) remained >10 in all our patients, and thus supplementation with an aromatase inhibitor was not required in any patient.

**Table 3:** Treatment characteristics and outcomes of patients HCG - human chorionic gonadotrophin; IU - international unit; FSH - follicle-stimulating hormone; TESA - testicular sperm aspiration

Case No.	Treatment regimen			Duration of treatment (months)	Sperm retrieval	Sperm freezing	Pregnancy
	HCG (IU) (initial/final)	FSH (IU) (initial/final)	Aromatase inhibitor				
1	2000/4000	75/150	NO	6 months	Yes (TESA)	NO	Positive
2	2000/5000	NO	NO	9 months	NO	NO	Negative
3	2000/5000	NO	NO	9 months	NO	NO	Negative
4	2000/5000	NO	NO	9 months	NO	NO	Negative
5	2000/5000	NO	NO	9 months	NO	NO	Negative
6	2000/5000	NO	NO	9 months	Yes (TESA)	YES	Negative
7	2000/5000	NO	NO	9 months	NO	NO	Negative
8	2000/5000	NO	NO	9 months	NO	NO	Negative
9	2000/5000	75/150	NO	5 months	Yes (TESA)	NO	Positive
10	2000/5000	NO	NO	9 months	NO	NO	Negative
11	2000/5000	NO	NO	9 months	NO	NO	Negative
12	2000/5000	NO	NO	9 months	NO	NO	Negative
13	2000/5000	NO	NO	9 months	NO	NO	Negative
14	2000/5000	NO	NO	9 months	NO	NO	Negative
15	2000/5000	NO	NO	9 months	NO	NO	Negative
16	2000/5000	NO	NO	9 months	NO	NO	Negative
17	2000/5000	NO	NO	9 months	NO	NO	Negative
18	2000/5000	NO	NO	9 months	NO	NO	Negative
19	2000/5000	NO	NO	9 months	NO	NO	Negative
20	2000/5000	NO	NO	9 months	NO	NO	Negative

Sperm freezing was required in only one patient, i.e., case no. 6. Sperm was successfully retrieved by testicular sperm aspiration (TESA) in three out of 20 patients. This yields a sperm retrieval rate of sperm as 15% in our study patients with NOA. All three patients showed late maturation on FNAC.

Table 4 shows the association of successful sperm extraction with histopathology. This association of sperm extraction with late maturation arrest compared to other histopathological findings was found to be highly significant, with a p-value of <0.001. Thus, hormonal treatment in patients with NOA seems to be beneficial only in patients with late maturation arrest of the gamete.

**Table 4:** Association of successful sperm extraction with histopathology Data was presented as n (%). Fisher's exact test was used to obtain the level of significance. The p-value was considered significant at less than 0.05 FNAC - fine needle aspiration cytology

FNAC	Sperm extraction successful		Significance
	Yes	No	
Early maturation arrest	0	4 (100%)	<0.001
Late maturation arrest	3 (100%)	0	
Hypospermatogenesis	0	5 (100%)	
Sertoli cell only	0	8 (100%)	

**Figure 1:**
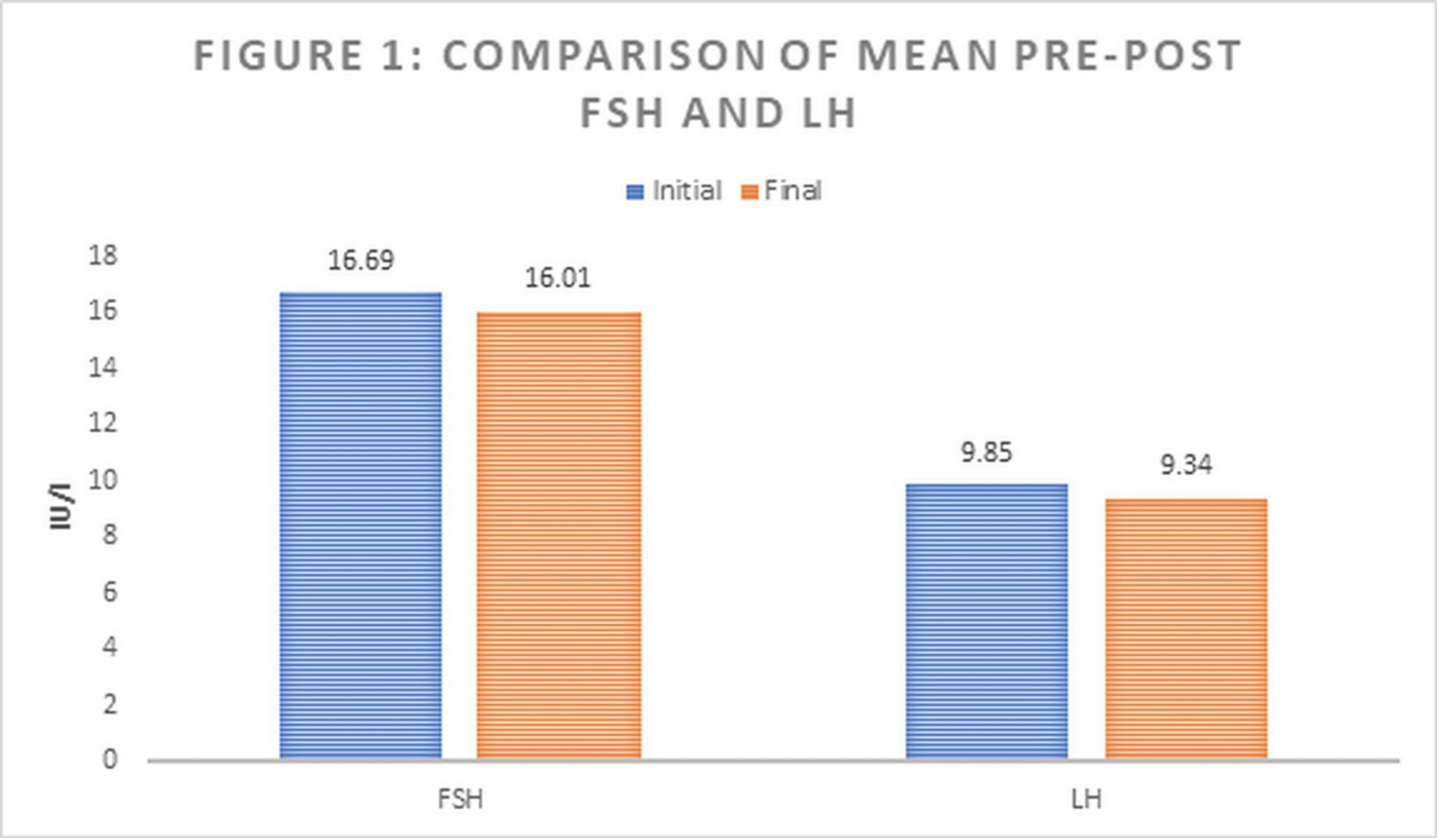
Comparison of mean pre- and post-levels of follicle-stimulating hormone (FSH) and luteinizing hormone (LH)

Figure 1 shows a comparison of the initial and final serum FSH and LH levels of all patients. The mean FSH level decreased from 16.69 ± 6.02 to 16.01 ± 7.32 IU/l with a p-value of 0.304, while the mean LH level dropped from 9.85 ± 1.95 to 9.34 ± 1.88 with a p-value of 0.192.

The comparison of the difference between initial FSH and LH levels across the two groups with successful sperm retrieval and unsuccessful retrieval was also studied. Table 5 shows a comparison of differences between initial FSH and LH levels across the two groups.

**Table 5:** Comparison of the difference between initial FSH and LH levels across the two groups Data was presented as mean±SD. An Independent t-test was used to evaluate the p-value. The p-value was considered significant at less than 0.05 FSH - follicle-stimulating hormone; LH - luteinizing hormone

Hormone	Sperm retrieval		p-value
	Yes	No	
FSH	2.77±2.63	0.31±2.86	0.185
LH	1.10±3.46	0.80±1.17	0.442

**Figure 2:**
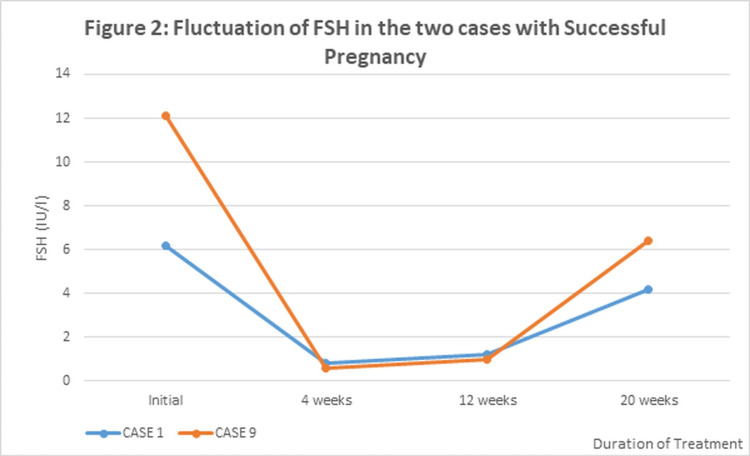
Fluctuation of follicle-stimulating hormone (FSH) in the two cases with successful pregnancy

**Figure 3:**
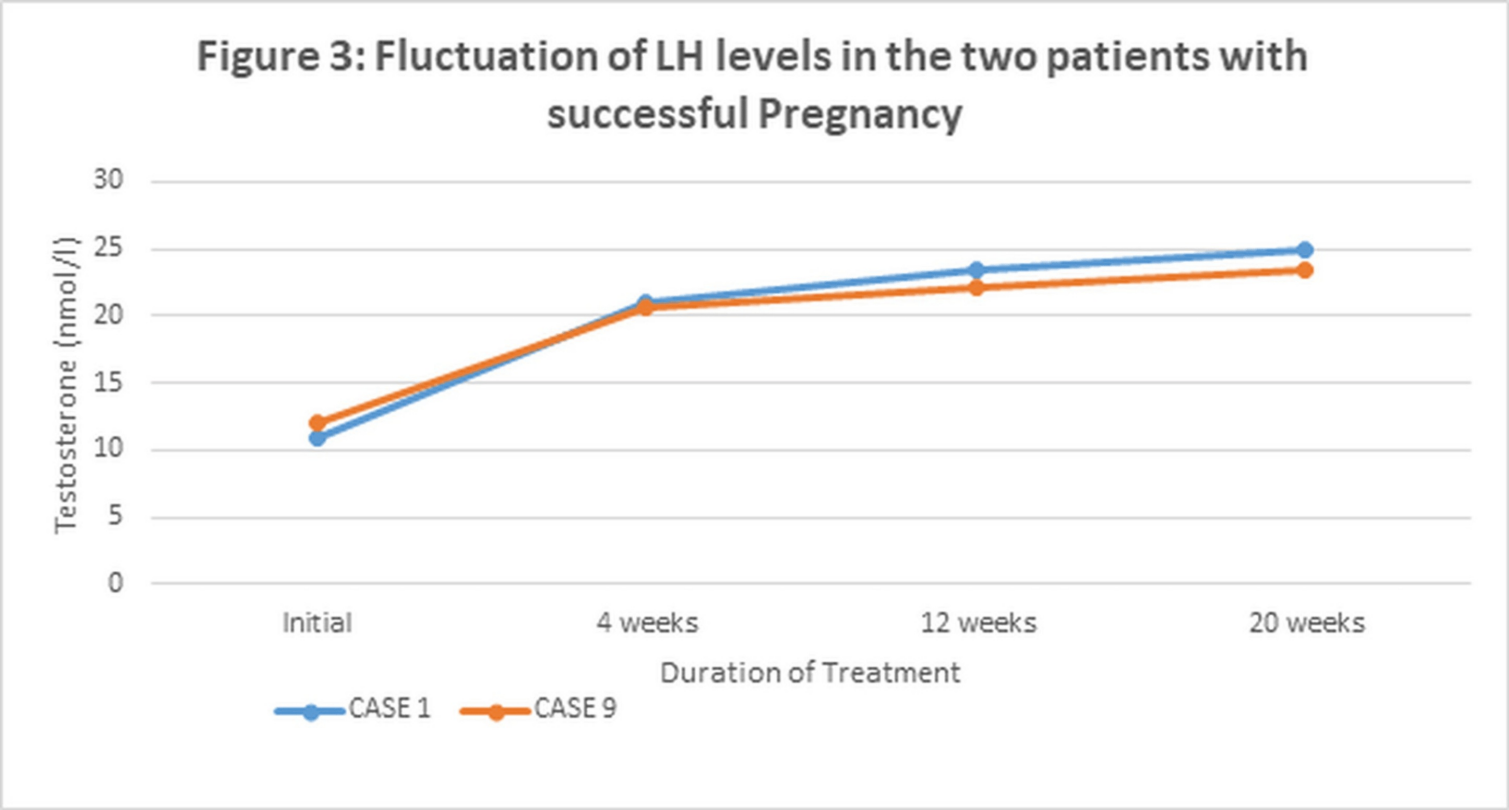
Fluctuation of luteinizing hormone (LH) levels in the two patients with successful pregnancy

Of the three patients in whom sperm retrieval was successful, subsequent pregnancy by intracytoplasmic sperm injection (ICSI) resulted in two patients. The trend of hormonal fluctuations for FSH and Testosterone in these two patients, in case 1 and case 9, where pregnancy was successful, is shown in Figure 2 and Figure 3, respectively.

Both cases showed a steep drop in serum FSH levels at four weeks after the initiation of gonadotropins. At four weeks, FSH was supplemented as per the treatment protocol, and it was observed that FSH remained stable for up to 12 weeks and then showed a gradual rise (Figure 2). On the other hand, serum testosterone levels showed an early increase in both patients at four weeks and then tended to remain stable thereafter. Figure 3 depicts fluctuations of LH levels in two patients of cases 1 and 9, respectively, with successful pregnancy.

## Discussion

Our prospective study showed the effect of gonadotropin therapy in 20 patients with NOA. It showed viable sperm retrieved by TESA in three cases. Two out of three cases (in whom sperm could be retrieved) have an ongoing pregnancy by ICSI. No natural conception could be achieved. Santi et al. reported improvement in semen parameters in 50% of patients [7]. In two short meta-analyses evaluating spontaneous pregnancy following gonadotropin therapy, the odds ratio (OR) for treatment was 4.94 and 4.5 [8,9]. Our study showed hypospermatogenesis, maturation arrest, and Sertoli cell only as the cause of NOA by Testicular FNAC. The sperm could be harvested only from patients with late maturation arrest.

In previous studies, positive results from gonadotropin therapy could be seen only in hypospermatogenesis and sperm maturation arrest [10]. This result is in concordance with our study's findings. According to earlier research, NOA frequently manifests as hypospermatogenesis and maturation stoppage. A study that was conducted in 2021 with 918 participants revealed that testicular biopsies performed during micro testicular sperm extraction (TESE) resulted in either pure or mixed hypospermatogenesis in 16.6% of cases and maturation arrest in 80.6% of cases [11].

In our study, two out of three cases had to harvest sperm in case of achieving pregnancy. The pooled clinical pregnancy rate was 39%, and 24% of couples experienced a live baby delivery in a recent systematic analysis of studies assessing pregnancy outcomes by ICSI utilizing sperm recovered by micro-TESE [12]. Both cases with ongoing pregnancies had positive results in the first ICSI attempt. Nevertheless, a previous case report described the development of a seminoma after prolonged FSH and HCG therapy [13].

Recombinant technology has met the requirement for a more dependable supply of FSH and hCG, especially in the treatment of female infertility, notwithstanding the aforementioned problems. Human FSH and HCG genes are usually used in the production process. These genes are inserted into the host cell's nuclear deoxyribonucleic acid (DNA) by a plasmid vector utilizing spliced DNA strings that contain the gonadotropin gene and pieces of bacterial DNA. Typically, recombinant gonadotropins are supplied as ready-to-use solutions in pen devices, which improves patient compliance with treatment by allowing subcutaneous injection [14,15].

Limitations

One of the limitations was the sample size, which might be due to convenience sampling. Another limitation can be the duration of the study. Also, the study was single-centric, which results in heterogeneous populations being affected. Thus, the control group was not enrolled in the study due to the small sample size and shorter duration.

## Conclusions

Our study concluded that gonadotropin therapy is helpful in very selected cases of nonobstructive azoospermia. For men with NOA and spermatogenic failure, recombinant gonadotropin therapy appears to be a useful method of overcoming infertility. The result could even be better if done in an appropriate case selection and using micro-TESE. Also, it could be noted that gonadotropin therapy should be considered for selected cases of NOA as an alternative to sperm donation. However, a larger population and multicentric study are required to further validate its efficacy and safety.
